# Supplementary material for: Conditional chemoconnectomics (cCCTomics) as a strategy for efficient and conditional targeting of chemical transmission
Source: eLife. 2024 Apr 30;12:RP91927. doi: 10.7554/eLife.91927 (PMC11060718; doi:10.7554/eLife.91927)
Supplement: Figure 3—source data 3. [file elife-91927-fig3-data3.zip › Figure 3 source data3 part 2.zip!/Figure 3-Source data 3-part2/1053f2-1-CRISPResso_Report_y3hlioj1.zip!/Nucleotide_percentage_quilt.pdf]

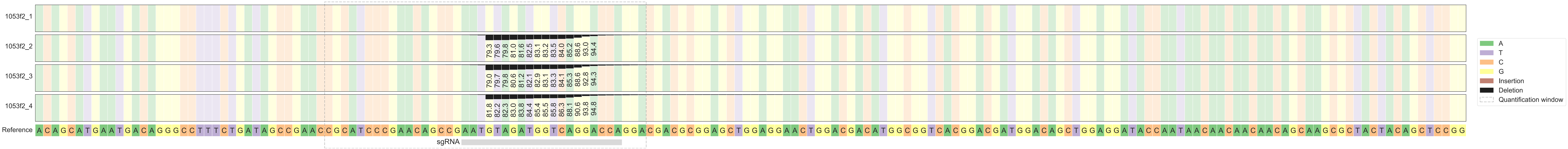
1053f2_1
1053f2_2
79.3 79.6 79.8 81.0 81.6 82.5 83.1 83.2 83.5 84.0 85.2 88.6 93.0 94.4
1053f2_3
79.0 79.7 79.8 80.6 81.2 82.1 82.9 83.1 83.3 84.1 85.3 88.6 92.8 94.3
1053f2_4
81.8 82.2 82.3 83.0 83.8 84.4 85.4 85.5 85.8 86.3 88.1 90.6 93.8 94.8
Reference
ACAGCATGAATGACAGGGCCTTTCTGATAGCCGAACCGCATCCCGAACAGCCGAATGTAGATGGTCAGGACCAGGACGACGCGGAGCTGGAGGAACTGGACGACATGGCGGTCACGGACGATGGACAGCTGGAGGATACCAATAACAACAACAACAGCAAGCGCTACTACAGCTCCGG
sgRNA
A
T
C
G
Insertion
Deletion
Quantification window
